# Local Bladder Cancer Clusters in Southeastern Michigan Accounting for Risk Factors, Covariates and Residential Mobility

**DOI:** 10.1371/journal.pone.0124516

**Published:** 2015-04-09

**Authors:** Geoffrey M. Jacquez, Chen Shi, Jaymie R. Meliker

**Affiliations:** 1 Department of Geography, University at Buffalo, The State University of New York, Buffalo, New York, United States of America; 2 BioMedware Inc., Ann Arbor, Michigan, United States of America; 3 Department of Preventive Medicine and Graduate Program in Public Health, Stony Brook University, Stony Brook, New York, United States of America; Kagoshima University Graduate School of Medical and Dental Sciences, JAPAN

## Abstract

**Background:**

In case control studies disease risk not explained by the significant risk factors is the unexplained risk. Considering unexplained risk for specific populations, places and times can reveal the signature of unidentified risk factors and risk factors not fully accounted for in the case-control study. This potentially can lead to new hypotheses regarding disease causation.

**Methods:**

Global, local and focused Q-statistics are applied to data from a population-based case-control study of 11 southeast Michigan counties. Analyses were conducted using both year- and age-based measures of time. The analyses were adjusted for arsenic exposure, education, smoking, family history of bladder cancer, occupational exposure to bladder cancer carcinogens, age, gender, and race.

**Results:**

Significant global clustering of cases was not found. Such a finding would indicate large-scale clustering of cases relative to controls through time. However, highly significant local clusters were found in Ingham County near Lansing, in Oakland County, and in the City of Jackson, Michigan. The Jackson City cluster was observed in working-ages and is thus consistent with occupational causes. The Ingham County cluster persists over time, suggesting a broad-based geographically defined exposure. Focused clusters were found for 20 industrial sites engaged in manufacturing activities associated with known or suspected bladder cancer carcinogens. Set-based tests that adjusted for multiple testing were not significant, although local clusters persisted through time and temporal trends in probability of local tests were observed.

**Conclusion:**

Q analyses provide a powerful tool for unpacking unexplained disease risk from case-control studies. This is particularly useful when the effect of risk factors varies spatially, through time, or through both space and time. For bladder cancer in Michigan, the next step is to investigate causal hypotheses that may explain the excess bladder cancer risk localized to areas of Oakland and Ingham counties, and to the City of Jackson.

## Introduction

With over 350,000 new cases each year, bladder cancer ranks ninth globally in incidence among all cancers [1]. Populations residing in industrialized areas in the U.S. and Western Europe have the highest incidence, with most cases diagnosed as bladder cancer transitional cell carcinomas (TCC) [2]. Occupational exposures and cigarette smoking are the major risk factors for bladder cancer, with some evidence implicating exposure to inorganic arsenic as a bladder cancer carcinogen [3]. But many cases remain unexplained.

The exposome has been defined as the totality of exposures over an individual’s life course, and quantification of aspects of the exposome relevant to specific health outcomes, such as bladder cancer, is difficult [4]. One approach is to consider unexplained risk. For example, case-control studies seek to determine whether certain factors are associated with increased disease risk among a designed sample of cases and controls. The disease risk not explained by those factors found significant in the case control study is the unexplained risk. Allocating that risk to specific local populations, places and times can reveal excesses that may be the signature of unidentified risk factors or risk factors that were not fully accounted for in the original study design. This may reveal localized exposures and behaviors, potentially leading to new hypotheses regarding disease causation.

Q-statistics have been developed as a method for accomplishing a decomposition of the unexplained risk into local populations, places and times [5,6]. This approach has been evaluated in simulation studies [7] and has been applied to testicular cancers [8], diabetes and leukemia [9], non-Hodgkins lymphoma [10], and breast cancer [11]. In this study we evaluate space-time patterns of bladder cancers in southeastern Michigan accounting for residential mobility and known risk factors and covariates. This research builds on a recently published population-based case control study of bladder cancer that used residential histories and information on occupational and life-style exposures to assess bladder cancer risks (The “parent study”) [12].

### Aims and Objectives

The overall objective of this study is to assess focused clustering of bladder cancer cases in southeastern Michigan. This study is motivated by several unmet methodological and applied challenges in geospatial methods and the epidemiology of bladder cancer. First, space-time patterns in bladder cancer accounting for residential mobility and adjusted for risk factors and covariates have yet to be studied and are largely undocumented. These patterns can provide important evidence potentially leading to novel etiological hypotheses. Second, the method of focused Q-statistics has never been applied to a complete case-control data set for any disease or health outcome. Addressing this need would be an important advance in applied methodology. Finally, an approach that combines information on temporal trends and persistence in the probability of Q-statistics has yet to be developed, yet would provide an important mechanism for evaluating potential false positives. This study aims to address each of these needs using existing Q-statistics, by developing new approaches for assessing temporal trends and persistence, and by applying these to a population-based case-control study of bladder cancer.

## Materials and Methods

### Ethics Statement

The University of Michigan IRB approved the parent case control study that enrolled participants, collected biological and drinking water samples, conducted surveys, and obtained the required written consent of study participants. Western IRB approved the analysis of secondary data from the parent study, which did not involve contact with study participants.

### Data

The data come from a population-based bladder cancer case-control study conducted in an 11 county area of southeastern Michigan. Designed to evaluate exposure to low levels of arsenic in drinking water as a risk factor for bladder cancer, this parent study evaluated a host of occupational and behavioral risk factors and covariates. We summarize the study design and sample characteristics of the parent studies, for details consult the original studies [12,13,14].

Incident cases of urinary bladder cancer diagnosed between 2000–2004 (411 cases) were recruited from the state cancer registry of the Michigan Cancer Surveillance Program. To the best of our knowledge, no more than 1 case occurred in a single family. The Michigan Public Health Institute (MPHI) recruited 566 controls that were frequency-matched to the recruited cases by gender, age (± five years), and race using random digit dialing of age-weighted lists. Age-weighted lists purchased from Genesys Sampling Systems were weighted to be representative of the age distribution of cases in the study area and were generated from telephone directories, automobile and motorcycle registries, real estate listings, and driver’s license data. This sampling frame does include the possibility of cell-phone users. Random calls were made to 11,463 potential controls, and eligibility was determined based on answers to screening questions (at least 5 years of residence in the study area, no history of cancer except non-melanoma skin cancer, and appropriate case-matched frequencies of age, race, and gender). Of those numbers dialed, 3,341 were non-working/non-residential or were never answered, 3,333 resulted in hang-ups prior to screening, and 4,748 resulted in successful screening. Of those screened, 2,616 were found to be ineligible. Among the 2,132 eligible controls, 69% refused to participate, 4% failed to complete all requirements of participation, and 27% completed all phases of participation including telephone interview, in-person interview, and providing environmental and biological samples, resulting in 566 participating controls.

There were 1,634 potentially eligible cases. Approximately 22% died prior to contact; the registry was not permitted by hospital or physician to contact another 5% of cases. The remaining cases were mailed a letter by the registry asking for permission to release their name and contact information to the research team. Of these 1,178 cases, 50% agreed to have their name released. Among the 584 cases subsequently contacted by the research team, 411 cases (70%) completed all phases of participation. Thus, of the 1,634 potentially eligible cases, 25% completed all phases of participation, resulting in 411 participating cases.

A 30–45 minute computer-aided telephone interview obtained information on smoking, medical history, diet, water and other fluid consumption. Residential and occupational histories were obtained through survey forms reviewed at home with each participant. Residential Addresses were geocoded using ArcGIS (Version 9.0; ESRI, Redlands, CA, USA) with geocoding parameters minimum match score 60, minimum candidate score 10, and spelling sensitivity 70. Geocoding accuracy was similar for cases and controls. 54% of controls’ person-years were geocoded to exact address or closest cross streets, and 11% geocoded to town center. For cases, 53% were geocoded to exact address or closest cross streets, and 14% geocoded to town center. Remaining residences were located outside the study area and were not geocoded.

Distributions of residential histories did not differ for cases and controls. Cases averaged 9.1 residences per person, controls averaged 9.0 residences. A total of 8,823 residences accounting for 64,040 person-years were reported in aggregate, with an average of 65 years of residential history per person. Participants spent 66% of their person-years within the study area.

The case-control study was originally designed to investigate the association between exposure to arsenic in drinking water and bladder cancer. Extensive effort went into estimating arsenic exposure over the life course for each participant, accounting for residential mobility patterns and changes in arsenic levels in private and public water supplies. For more details refer to Meliker et al (2007) [15]. Time-weighted average lifetime exposure to arsenic was calculated and treated both as a continuous variable, and categorized a priori into <1 μg/L, 1–10 μg/L, and >10 μg/L [12].

Unadjusted logistic regression analyses were conducted, as were analyses adjusted for covariates age, race (white, black, other), and sex, and for bladder cancer risk factors found significant or borderline significant: smoking (never smoker, former < 20 pack-years, former ≥ 20 pack-years, current < 20 pack-years, current ≥ 20 pack-years), education (highest level attained), history of urinary bladder cancer in an immediate relative (parent, sibling, or child), arsenic in drinking water, and at least five years of employment in a high risk occupation (dye workers and users, aromatic amine manufacturing, leather workers, painting, driving trucks or other motor vehicles, aluminum workers, machinists, and automobile assemblers). As groups, cases and controls did not differ by gender, race, and age, nor by average age of 65 years. Cases smoked more cigarettes over their lifetime, completed fewer years of schooling, and were more likely to have worked for at least five years in an occupation at high risk for bladder cancer; they also were more likely to have a family member diagnosed with bladder cancer. The variables found to be significant bladder cancer risk factors, and the corresponding logistic regression models from the parent study, were used in our research. Further details on case ascertainment, recruitment, random digit dialing, odds ratios, and study design are published in the parent studies [12,13,14,15].

### Models and Statistical Methods

#### Q statistics

Jacquez et al. [5,6] develop global and local tests for case-control clustering of residential histories, using an algebraic discrete time representation. Here we use the continuous time representation [7] and the multiple correction techniques that support identification of etiological hypotheses [11].

Q-statistics are based on a space-time step function that documents a person’s residential mobility over the life course. This is quantified using a matrix representation that measures how geographic nearest neighbor relationships change through time. Q-statistics for assessing various types of space-time clustering are available, for overall global clustering, for spatial and temporally local clustering, for clustering at specific time intervals, and for assessing focused clustering about point sources. These have several desirable properties, the first being that the global tests can be decomposed into local tests such that the sum of the local tests yields the global test statistic. Here we use local, global and focused tests summarized below, using the notation developed and applied in earlier publications. For methodological details refer to these prior publications [6,7,11,16].

The local statistic from which all other tests are derived is called “Q_it_” and is

$$Q_{i,t}^{\left(k\right)}=c_{i}\sum_{j=1}^{k}\eta_{i,j,t}^{\left(k\right)}c_{j}.$$ 1

This is the count, at time *t*, of the number of *k* nearest neighbors of participant *i* that are cases, and not controls. The variables *c*_*i*_ and *c*_*j*_ are case-control identifiers, and $\eta_{i,j,t}^{\left(k\right)}$ is a nearest neighbor indicator. Simulation studies demonstrated good statistical power when a *k* of 15 is used [7], the parameter value employed in this study.

A life-course, subject-specific statistic that integrates through time is “Q_i_” and is calculated as:

$$Q_{i}^{\left(k\right)}=\int_{t=t_{0}}^{T}Q_{i,t}^{\left(k\right)}\;dt.$$ 2

This statistic assesses a tendency to have other cases, rather than controls, nearby over the life course of participant *i*. A time-specific statistic that provides an overall measure of case clustering when all of the participants are considered together is “Q_t_”:

$$Q_{t}^{\left(k\right)}=\sum_{i=1}^{n_{1}}Q_{i,t}^{\left(k\right)}.$$ 3

Q_t_ is the sum at time *t*, over all cases, of the Q_it_ (the subject-specific and time-specific measure of case clustering in Eq 1). $Q_{t}^{\left(k\right)}$ evaluates global spatial clustering of cases at time *t*. A global clustering statistic that considers clustering of cases for all of the cases and controls, over the entire study period, is “Q” and is:

$$Q^{\left(k\right)}=\sum_{i=1}^{n_{1}}Q_{i}^{\left(k\right)}.$$ 4

Focused statistics quantify clustering around a specific location or focus. Lawson (1989) [17] and Waller *et al*. (1995) [18] proposed focused tests that do not account for human mobility, nor for mobility of the focus itself.

A focused Q statistic for clustering of cases about a focus at time *t* is:

$$Q_{F,k,t}=\sum_{j=1}^{N}\eta_{F,j,k,t}\;c_{j}.$$ 5

The nearest neighbor index *η*_*F*,*j*,*k*,*t*_ indicates whether the *j* ^th^ individual is a *k* ^th^ spatial nearest neighbor of focus **u**_*F*,*t*_ at time *t*. The statistic *Q*_*F*,*k*,*t*_ is the count, at time *t*, of the number of *k*-nearest neighbors of the focus that are cases. A test for focused clustering through time is:

$$Q_{F,k}=\sum_{t=0}^{T}Q_{F,k,t}.$$ 6

Over T times, this is the count of the number of cases that are *k* nearest neighbors of the focus at each time point.

In this paper, we use Eq 1 ($Q_{i,t}^{\left(k\right)}$) to identify when and where an individual is a center of a local cluster. We use Eq 2 ($Q_{i}^{\left(k\right)}$) to identify which individuals tend to be centers of clusters over their life-course. We use Eq 3 ($Q_{t}^{\left(k\right)}$), and plots of this probability of $Q_{t}^{\left(k\right)}$ through time, to identify time periods with significant case clustering. We use Eq 4 (global *Q* ^(*k*)^) to assess whether global clustering exists when the residential histories of all of the participants are considered over the entire duration of the study. We use Eq 5 (*Q*_*F*,*k*,*t*_) to assess clustering of cases about focus F at time *t*, and Eq 6 (*Q*_*F*,*k*_) to quantify clustering about focus *F* over time. Finally, we use a Global Q_F_ that is calculated as the sum of the *Q*_*F*,*k*_ over the *F* foci to assess global focused clustering when all foci are considered over the entire time period of the study. Refer to earlier publications for implementation details on Q-statistics [6,11,16].

The remainder of this paper drops the “*k*” notation, writing Q_*it*_ for the local statistic; Q_*i*_ for the subject-specific life-course statistic; Q_*t*_ for the time-specific large-scale spatial cluster statistic and so on dropping “*k*” notation for the focused statistics in a similar fashion.

#### Inferential framework and multiple testing correction

Space-time pattern recognition techniques can provide insights into specific etiologic hypotheses [7]. Further, considering sets of significant Q-statistics both accounts for multiple testing and supports process-based inference [11]. This study employs sets of significant Q-statistics for both multiple testing corrections and to construct inferences regarding etiological hypotheses, see the earlier publications cited above for details.

The “raw” Q-statistics, especially the local statistic *Q*_*it*_, are quite useful for identifying local risk excesses. However, there are many such local statistics and one must correct for multiple testing. Traditional approaches such as the Bonferroni correction and sequential methods like Simes-Hochberg [19,20] and Hommel’s method [21] are widely recognized as too conservative [22] We use an approach that is disease process and pattern oriented [7,11]. We employ the significance of the number of elements (a cardinality statistic) in each of the cluster sets. When significance of the number of elements in a cluster set is demonstrated one then inspects the p-values of the constituent local tests to identify those local statistics that contributed the most to the overall significance.

#### Inference from trends and persistent local clusters

Time trends in the probability of cluster statistics may be used to assess (1) overall temporal patterns in spatial measures of clustering, as these may indicate the long-term influence of causal factors; and (2) the persistence of local spatial clusters of cases. The logic behind this may be seen from a Gedanken experiment. Suppose there is no space-time pattern in the cases and controls, and then impose, at some time t_1_, a local or focused cluster small enough so that, while it causes significant local clustering, does not result in significant global clustering. By definition, the global cluster statistic is the sum of the local cluster statistics; the global statistic thus must increase after time t_1_, and its associated probability must decrease. Hence persistence is key as we expect false positives to be ephemeral. Finally, sound inference requires evaluation of a biologically plausible mechanism. For example, persistent clustering about a focus would require a plausible exposure from the industrial activities associated with that focus. Hence inspection of temporal trend in the probability of local statistics, coupled with assessment of local cluster maps, provides a tool for assessing the signature of local excess disease risk.

#### Randomization and null hypotheses

The significance of the Q statistics is assessed through randomization of case-control identifiers over the mobility histories. We use two null hypotheses. The first is no association between places of residence and case-control status—we call this “not adjusted”. Under “not adjusted”, each subject has a probability of being labeled a “case” in proportion to the ratio of cases to controls in the study. The second null hypothesis, called “adjusted” incorporates information on bladder cancer covariates and risk factors, and employs predicted probabilities of being a case calculated from logistic regression [6]. Here, each subject has a probability of being labeled a “case” in proportion to their probability under the logistic regression. We used either 999 or 9,999 randomizations; 999 initially as a screen and 9,999 to better resolve p-values found near the alpha level of the given test. 999 randomizations allowed us to resolve p-values as small as 0.001; 9,999 allowed us to resolve p = 0.0001.

#### Study Design

We ran both “not adjusted” and “adjusted” Q-analyses, using the logistic model for this data set as described in [12] for the adjustment model. This model included variables found significant (for both statistical and etiological reasons) and included arsenic exposures, education levels, smoking activities, family history of bladder cancer, occupational exposure to bladder cancer carcinogens, age, gender, and race. The model is expressed in terms of the probability of being a case given the risk factors and covariates for each individual. All variables included in the model and their corresponding coefficients are listed in Table 1. Hence any clustering found after adjustment thus is above and beyond that explained by those variables in the logistic model.

**Table 1:** The risk factors and covariates included in the logistic model and their corresponding coefficients.

Risk factors and covariates	Variables	Number of individuals	Coefficients
Estimate of average lifetime arsenic in drinking water	Less than 1 mg/L	452	-
	Greater than 1 mg/L and less than 10 mg/L	448	-0.1741
	Greater than 10 mg/L	77	0.1031
Education	no college	299	0.4136
	some post-HS training (not graduated from 4-yr institution)	268	0.2704
	college graduate or more years of schooling	410	-
Smoking activities	never smoker	360	-
	former smoker < 20 pack-years	210	0.4811
	former smoker 20+ pack-years	262	1.0694
	current smoker < 20 pack-years	19	-0.0113
	current smoker 20+ pack-years	121	1.4114
Family history of bladder cancer	Yes	37	0.6402
	No	940	-
Occupational exposure to bladder cancer carcinogens	Yes	147	0.3945
	No	830	-
Age ^a^	Age at time of interview / diagnosis	977	-0.00676
Gender ^a^	Male	733	-
	Female	244	0.000464
Race ^a^	White	898	-
	Black	26	-0.1199
	Other	53	-0.0597
Intercept	Intercept	-	-0.6736

Details on odds ratios, confidence intervals, risk factors and covariates, and arsenic exposure estimation are given by Meliker et al [12,13,14,15].

^a^ There was frequency matching on age, race, and gender which explains the small size of their coefficients.

We employed two temporal orientations, date and age. The date temporal orientation used the dates on which events transpired (e.g. diagnosis, residence relocation) when assessing space-time patterns. The age orientation used the age of the individual participant to record when these events transpired. The age orientation is useful for assessing clustering associated with critical ages in the life course, for example occupational clusters that occur in working years, and those associated with critical periods of vulnerability. Date orientation is sensitive to clustering attributable to exposures, for example, associated with specific time periods, such as the operations of polluting industries.

For the focused analyses we used the business address histories and years of operation of industries in south east Michigan whose activities used or produced compounds that are known or suspected bladder cancer carcinogens. We constructed a database of 268 industries using the Toxics Release Inventory [23] and the Directory of Michigan Manufacturers for years 1946, 1953, 1960, 1969, 1977, and 1982. The start date was no earlier than 1943 for any industry (assuming those in the 1946 directory did not begin in 1946). For industries that opened up after 1946, the start date was defined as the midpoint between the first directory where they were present and the preceding directory (for example, 1965 as a start date for those industries that were not present in 1960 but were present in 1969). Similar methods were used for defining the end date.

Validation of the Q methods was accomplished using Cuzick and Edwards test [24], which is analogous to the Q_t_ statistic in that it yields a statistic quantifying global spatial case clustering at a fixed time point. We validated Q_it_ using exact permutation for small data sets. We also compared cluster results to the scan statistic Bernoulli model [25,26], noting this method is not sensitive to small clusters that can be detected using either Q or Cuzick and Edwards test [27].

We ran the Q statistics using the adjustment for the covariates and risk factors found significant by Meliker *et al*. (2010) [12], including arsenic exposure. We ran the not focused and focused Q statistics, yielding global cluster tests (e.g. Q and Q_F_), tests for case clustering at specific time points (e.g. Q_t_ and Q_Ft_), case clustering about specific cases and industries over time (Q_i_ and Q_Fi_), and local clustering about specific individuals or industries at time t (Q_it_ and Q_Fit_). We used the set intersection statistics of Jacquez et al (2014) [11] to account for multiple testing and for the inferential framework. We undertook these analyses using year and age-based orientation. We also inspected time plots showing trends in the probability of the numbers of significant Q_t_ and Q_FT_ along with cluster maps of p(Q_it_) and p(Q_Fit_) to identify significant local clusters that may not have been large enough to lead to global significance. All analyses were undertaken in SpaceStat [28].

## Results

The analysis results adjusted for multiple testing using the count of the number of significant tests with 999 randomizations are summarized in Table 2.

**Table 2:** Summary of analysis results of global tests with 999 randomizations.

	Q with calendar year 1940–2003^a^		Q with age^b^		Q_F_ with calendar year 1943–1999^c^	
	N^d^	P value^e^	N^d^	P value^e^	N^d^	P value^e^
Global Q, Q_F_	-	0.390	-	0.236		0.100
Life course ${\tilde{Q}}_{i}$, ${\tilde{Q}}_{Fi}$	13	0.842	18	0.543	19	0.186
Time ${\tilde{Q}}_{t}$, ${\tilde{Q}}_{Ft}$	3	0.480	6	0.269	29	0.216
Local ${\tilde{Q}}_{it}$, ${\tilde{Q}}_{Fit}$	2566	0.778	283	0.659	1353	0.085
Life course and local ${\tilde{Q}}_{i}\&{\tilde{Q}}_{it}$, ${\tilde{Q}}_{Fi}\&{\tilde{Q}}_{Fit}$	832	0.790	113	0.603	851	0.123
Time and local ${\tilde{Q}}_{t}\&{\tilde{Q}}_{it}$, ${\tilde{Q}}_{Ft}\&{\tilde{Q}}_{Fit}$	36	0.458	65	0.201	77	0.301
Life course, time and local ${\tilde{Q}}_{i}\&{\tilde{Q}}_{t}\&{\tilde{Q}}_{it}$, ${\tilde{Q}}_{Fi}\&{\tilde{Q}}_{Ft}\&{\tilde{Q}}_{Fit}$	18	0.404	10	0.343	0	1.000

^a^ Q statistics over the entire study period 1940–2003 using year-based measure of time.

^b^ Q statistics using age-based measure of time.

^c^ Q_F_ statistics about industries in operation from 1943 to 1999 using year-based measure of time.

^d^ The count of the number of clusters found significant at *α* ≤ 0.05.

^e^ The significance of the number of clusters found significant at *α* ≤ 0.05.

Overall, there is little suggestion of significant clustering when inspecting the statistical results in Table 2. These are based on the set intersection statistics that adjust for multiple testing and are all global tests based on the significance of the number of local statistics. We thus expect these to be sensitive to “big signals” that will have global impacts in terms of clustering over most of the cases in the study, either in space, in time, or through both space and time. For the regular (not focused) Q-statistics the analyses by age have smaller p-values, although none of these are significant. The focused tests overall have the smallest p-values, with a minimum achieved for the local variants of p = 0.085. We thus conclude there is little evidence of large-scale global clustering of bladder cancer cases through time. This does not preclude the possibility of significant local clusters whose signal is diluted by an otherwise random pattern in the remainder of the cases, localities and times considered. We therefore assess temporal trends in global spatial statistics, along with maps of significant local clusters, to identify persistent and geographically localized excess risk. In order to better resolve p-values found near the alpha level of local tests, 9,999 randomizations were conducted for the following analyses.

**Fig 1:**
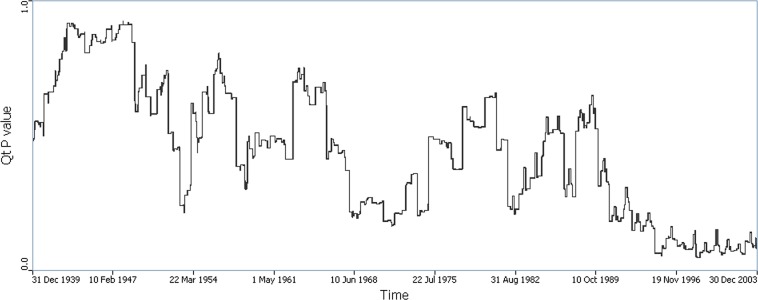
Time plot of p(Q_t_) using year-based measure of time. Minima on the time plot indicate time periods when global spatial clustering of cases were statistically significant.

The Q-statistics were run for the period 1940–2003. For the life-course statistic Q_i_, 14 cases were found statistically significant (S1 Fig). Among them, seven are in Ingham, four in Jackson and three in Oakland. In addition, a decreasing trend of Q_t_ p-values was observed (Fig 1). Q_t_ p-values in the period 1995–2003 were generally lower than 0.1 with the lowest (smaller than 0.05) being observed January—August, 1995 and September—December, 1998. However, the number of significant Q_i_ statistics was not significant overall, nor was it for Q_t_ (global spatial clustering of cases at time *t*).

**Fig 2:**
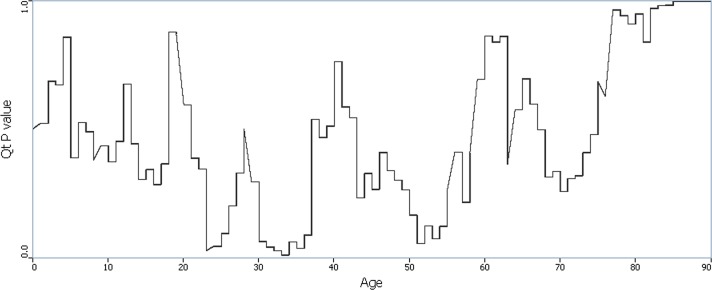
Time plot of p(Q_t_) using age-based measure of time. Probability of clustering of cases by age. Minima in this graph occur at ages that exhibit increased statistical evidence of clustering of cases at a given age relative to controls at that age.

Analyses were conducted for ages 1 year to 90 years old. Using the Q_i_ statistics, 20 cases were found statistically significant over their life course. The majority of these cases (14 out of 18) lived in Jackson (S2 Fig), and the remainder in Oakland, Genesee and Shiawassee. Additionally, the results of the Q_t_ analyses showed that two age intervals were associated with significant spatial clustering, namely, 23–24 and 31–35, as shown in Fig 2. The smallest p-values (smaller than 0.1) were also observed at the age of the early 50s. When adjusted for all years considered the number of significant Q_i_ statistics was not significant, nor was the overall number of significant Q_t_ statistics.

**Fig 3:**
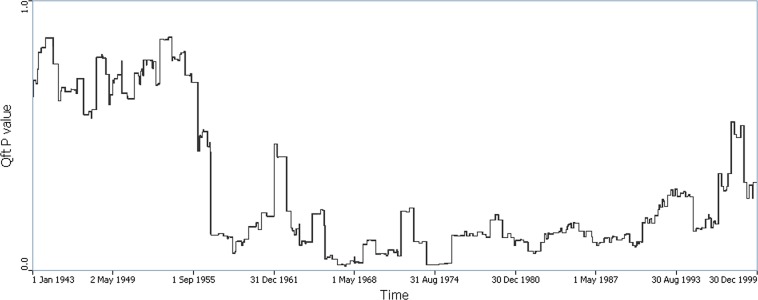
Time plot of p(Q_F_) using year-based measure of time. Values plotted are the probability of focused clustering when all of the industrial sites are considered simultaneously (e.g. probability of global focused clustering). The period of significant global focused clustering observed in 1974–1975 is attributable to the focused cluster that arose in the City of Jackson.

Focused statistics were calculated for the operational period of the industries (1943–1999). Using (Q_Fi_), out of 268 industries considered, 20 were identified as statistically significant focuses through the entire period (S3–S6 Figs). These were primarily in Ingham county near the city of Lansing (16 out of 20); others were found in Oakland county (4 out of 20) (see Table 3 for details). The time intervals associated with significant focused clustering were 1958, 1966–1968 and 1974–1975 (Fig 3). In the late 1950s (i.e., 1957–1959), the significance of Q_Ft_ dropped considerably coincident with a rapid increase in the number of industries in the industry database. The number of industries increased from 54 in 1956 to 87 in 1957; and the number of industries associated with significant Q_Ft_ statistics doubled, from 7 in 1956 to 15 in 1957; these were all in Ingham county.

**Table 3:** The 20 industries whose business address histories were found to be persistent centers of bladder cancer case clusters.

Business name	Address history	City	County	Start Year and End Year (Years in Operation)	Industrial activity engaged in	Estimate maximum # of employees
Kish Industries Inc	1301 Turner	Lansing	Ingham	1950–1965 (16)	Paint Mfg	100
Liquid Glaze Inc.	704 Sheridan	Lansing	Ingham	1943–1973 (31)	Paint Mfg	60
Michigan Co. Inc	412 N Washington	Lansing	Ingham	1950–1965 (16)	Chemical Mfg	?
Michigan State Solvents	1800 Glenrose	Lansing	Ingham	1957–1965 (9)	Chemical Mfg	10
Fibermark	340 Mill St.	Rochester	Oakland	1997–2003 (7)	Paper mill	?
Auto-Air Industries, Inc.	706 Sheridan	Lansing	Ingham	1957–1965 (9)	Plastics Mfg	37
Paracon Plastics Co.	702 Sheridan	Lansing	Ingham	1957–1965 (9)	Plastics Mfg	?
Bannasch Welding Inc.	602 N. Larch	Lansing	Ingham	1957–1965 (9)	Welding	5
Van Straaten Chemical Co.	501 N Walnut St	Lansing	Ingham	1957–1965 (9)	Chemical Mfg	?
Lapaco Chemical Inc.	1800 Glenrose	Lansing	Ingham	1943–1956 (14)	Chemical Mfg	38
Consolidated Industrial and Agriculautral Chemicals	2011 N. high St.	Lansing	Ingham	1957–1965 (9)	Chemical Mfg	?
Wheaton Chemical Co.	131 N. Pere Marquette D	Lansing	Ingham	1943–1965 (23)	Chemical Mfg	30
E-Z Flo Chemical Company	2011 High st.	Lansing	Ingham	1950–1973 (24)	Chemical Mfg	23
Vantico Inc.	4917 Dawn Ave	East Lansing	Ingham	1997–2003 (7)	Plastics Mfg	?
Dudley Paper Co.	740 E. Shiawassee	Lansing	Ingham	1957–1965 (9)	Paper Processing	14
Superior Paint Co.	High St	Lansing	Ingham	1943–1949 (7)	Paint Mfg	?
Screen art Inc.	1217 Turner Rd	Lansing	Ingham	1957–1978 (22)	Cotton Fabric Finishing	5
Thoreson-McCosh Inc.	1885 Thunderbird	Troy	Oakland	1979–1996 (18)	Paint Mfg	41
American Eagle	1130 E. big Beaver	Troy	Oakland	1974–1978 (5)	Cotton Fabric Finishing	5
Galan Mfg Co	2260 Scott Lake Rd.	Pontiac	Oakland	1966–1973 (8)	Tubing Fabrication	54

The majority of the industries in Table 3 were located in Lansing, Michigan and were manufacturers of paints, chemicals, or plastics. Similar industries are found elsewhere, however chemical manufacturing was more pervasive in Lansing, and continued for a longer period of time than in other parts of the study area. The prospect of environmental pollution originating from these chemical manufacturing facilities being associated with bladder cancer is intriguing, although identifying which chemicals were emitted by these industries during that time is difficult. Further, since the global statistics in Table 2 were not significant, the possibility of multiple testing and possibility of false positives should not be ignored when looking at the local cluster results.

It is worth noting that the local cluster analysis that identified the Lansing cluster coincides in some respects with the timing and location of the Ingham County focused cluster. This is to be expected as significant focused clustering can induce the finding of a local cluster in the vicinity of the focus. This (finding of coincident local and focused clusters) should not be taken as additional evidence of a focused cluster.

## Discussion

This study develops extensions and applications of Q-statistics aimed at finding significant clusters of cases accounting not only for risk factors but also for temporal dynamics and spatial location and mobility of the cases. Considering temporal dynamics and residential mobility is a step forward over other tests that consider spatial distributions only, or that do not account for mobility, such as Cuzick and Edwards test [24], the spatial and space-time scan tests, and others e.g. [26,29,30,31].

This research makes three important contributions. It is the first application of focused Q-statistics in a completed case-control study. Second, using temporal trends and persistence in the probability of Q-statistics to evaluate potential false positives, as summarized in the Gedanken experiment, is new. Finally, this is the first assessment of space-time patterns in bladder cancer using Q-statistics. An earlier methodological publication used incomplete data from the parent study (for which enrollment was ongoing at the time), and found global focused clustering [5]. Here, we analyzed the complete case-control using the full dataset, did not find global focused clustering, but did find temporally persistent and statistically significant focused clustering about specific industries.

This study has several salient limitations and caveats. First, the parent study itself was possibly subject to selection bias, recall bias, and a geographic bias in enrollment, these are discussed in detail in Meliker et al (2010) [12]. For this study, the possibility of an urban-rural bias in participation merits particular consideration. Meliker et al (2010) reported that participating cases were younger, having an average age of ~60 years in contrast with non-participating cases (average age ~70’s). In addition, cases who died prior to contact for enrollment were more likely to come from rural areas. It thus is possible deceased non-participating cases came from rural areas characterized by higher exposures that led to early death, thereby biasing focused tests towards finding clusters in urban areas. Second, geocoding was used to ascertain the residential histories of cases and controls, as well as industries. Positional uncertainty in geographic coordinates is currently not reported as part of geocoding protocols [32,33], and how this uncertainty changes through time is unknown. This problem is largely undocumented and unrecognized, although its potential impact on analysis results is potentially significant [34]. Should positional uncertainty increase through time the impact on cluster statistics likely would be increased bias towards the null finding of no clustering for earlier time periods. Finally, on-going applications of Q-statistics have used 2 or more control samples to provide an additional level of validation of cluster findings, but was not accomplished in this study.

Overall global significance of the regular and focused Q analyses was not observed, indicating a “big signal” consistent with large-scale geographic case clustering is absent. Global tests using age-orientation all have smaller p-values than the corresponding tests using year-orientation, suggesting age-based clustering may be acting for specific age subsets, but is not strong enough to generate age-based clustering globally when all ages combined are considered.

As noted earlier in the paragraph on the Gedanken experiment, it is possible for local clustering to be present but for significant global clustering to be absent. In this case one inspects time trends for the global statistics, and maps of local excesses, to evaluate whether there are persistent local clusters through time. This signature should be different from that expected for false positives whose presence has no causal basis (they are chance outcomes), and thus should be ephemeral and lack any biologically plausible exposure mechanism.

Trends in the time plots of the global statistics (Q_t_ and Q_Ft_) both reveal time periods with significant spatial case clustering. The question then arises as to the locations and timing of local clusters that might explain these observed smaller probabilities for age orientation, and the time periods of persistent significant clustering.

Results from the Q analyses using the not focused tests found geographically localized clustering that persists through time in Oakland and Ingham counties. This local clustering is sufficiently strong to cause the probability of the global statistic (Q_t_) to trend downward during the 1990’s and 2000’s, becoming statistically significant in 1995–2003. The persistence through time of the Oakland and Ingham clusters suggests the action of a localized risk factor not accounted for in the modeled risk factors and covariates. These clusters persist even after arsenic exposure is accounted for, and thus may be attributable to a geographically localized risk factor that has yet to be identified.

The overall decrease in the probability p(Q_t_) through time, and the downward trend in the time plot of p(Q_t_) is an interesting feature that plausibly might be explained by changing data quality (e.g. accuracy and completeness) of the place of residence data through time. Place of residence information was obtained by survey, and it is likely that residential addresses from many years ago are less likely to be recalled accurately, and may also be difficult to geocode (due, for example, to time-dependent mismatch in the street address database used by the geocoding engine). This increased uncertainty in geocoded place of residence for earlier addresses may lead to decreased accuracy and completeness in the geocoded coordinates. This should make it more difficult to detect any true cluster pattern that may exist, although to our knowledge at this time of writing studies have yet to be conducted that assess impacts of time-dependent uncertainty in address histories with the statistical behavior of cluster tests.

The results from the focused cluster analyses in general corroborate the results from the not focused tests, but with important differences. Geographically, the focused cluster analysis agreed well with the not focused analyses that used the calendar year orientation, although subtle differences were still present. The cluster in the city of Jackson observed for ages in the 20’s and mid 30’s using the not-focused statistics were echoed by the focused analyses that found statistically significant industries in Jackson city. Temporally, the time trends in p(Q_t_) and p(Q_Ft_) were different. Specifically, we do not observe the consistent downward trend in p(Q_Ft_) that we observe in p(Q_t_). This is not unexpected, as Q_Ft_ and Q_t_ are sensitive to different aspects of spatial clustering of cases.

We identified 20 industries that were the center of persistent, focused clustering of cases. Each of these was involved in industrial activities involving compounds or processes that are known or have been implicated as bladder cancer carcinogens. It must be emphasized that possible exposure mechanisms were not identified or explored in this study; biomarkers of exposure have not been assessed for any of the cases involved in such clusters; nor have any releases of such bladder cancer carcinogens from these 20 sites been documented or measured. Furthermore, statistical significance of the global focused clustering statistic was not found. While we did observe a decrease in the probability of the temporally local focused cluster statistic Q_Ft_ when these industries began operations, this of itself does not implicate these industries as a cause of bladder cancer cases in the Lansing area. In the absence of such information the findings of significant focused clustering in the vicinity of these industries cannot be attributed to business activities or industrial processes undertaken by these industries.

One might consider undertaking an adjustment for major industries in the neighborhood of study subjects. Might such an adjustment explain the observed cluster? We do not know of a statistical approach that, in the absence of plausible exposure measurements and/or transport and fate models, can satisfactorily accomplish individual-level adjustment for major industries in the neighborhood of study subjects. Instead, our approach adjusts for known risk factors and covariates, and then asks whether there is significant focused clustering of the unexplained risk. Here, the focused cluster model posits that cases cluster about industrial sites that employ processes that involve known or suspected bladder cancer carcinogens. Once such clustering is demonstrated, an exposure assessment for those industries that are known cluster foci may be warranted.

The age-based analysis yields qualitatively different results from those obtained using the year orientation. As noted earlier, a strong, geographically localized cluster is apparent in the vicinity of Jackson City, occurring in those ages consistent with an occupational exposure or a geographically localized risk factor operative during an individual’s 20’s and 30’s. It is noteworthy that the late 20’s did not show significant clustering in Jackson, although the early 20’s and early 30’s did.

The number of cases and controls extant in the study area changed through time, both seasonally as “snow-birds” left for the winter months, and then returned; and also over the course of the study, as study participants were born or moved into the study area. We inspected not only the total number of study participants through time, but also how the ratio of cases and controls varied. The case-control ratio did not change appreciably through time; however, the total number of participants was 194 in 1940, 316 in 1950, 639 in 1970, 500 in 1990, and 974 in 2003 and increased thereafter. As the sample size decreases the variance of Q-statistics is expected to increase, making detection of true space-time excesses more difficult at smaller sample sizes. To date, little research has been conducted on the impacts of changing sample size on the statistical power of space-time cluster statistics.

We also conducted analyses using models of the Empirical Induction Period [16]. We found these statistics to be sensitive to changes in the proportions of cases and controls extant during the time periods under consideration. This suggests when using latency models to detect clustering that one may wish to specify a null hypothesis that is not sensitive to time-based changes in the proportion of cases, unless this is a feature the analyst wishes to detect.

This paper illustrates how Q analyses combining time geography and founded on a sound case-control study design provides a powerful tool for unpacking unexplained disease risk. This is especially useful when the design of the parent case-control study did not include important risk factors whose action varies spatially, through time, or through both space and time. This makes possible the re-analysis and mining of completed case-control studies to generate new hypotheses and elucidate space-time patterns of disease risk. For bladder cancer in Michigan, the next step is to identify specific risk factors that may underlie the excess bladder cancer risk that has been localized to areas of Oakland and Ingham counties, and to the City of Jackson.

## Supporting Information

S1 Fig Cluster map of p(Q_it_) for October 1, 1998. Maps of the significant local space-time statistic p(Q_it_) illustrate the persistent local bladder cancer clusters in Oakland county, Ingham county near Lansing, and in the city of Jackson. Visualized for October 1, 1998.
(TIF)

S2 Fig Cluster map of p(Q_it_) for 33 year olds. The Jackson cluster constructed using place of residence of cases and controls at age 33 years.
(TIF)

S3 Fig Cluster map of p(Q_Fit_) for June 1, 1956. Red squares indicate industrial sites that are the foci of significant case clustering at the 5% of smaller level. The group of significant foci in the Lansing area are responsible for the large decrease in the global cluster statistic Q_F_ that began in 1956 and 1957, and persisted through 1964. By 1967 this cluster was no longer significant.
(TIF)

S4 Fig Cluster map of p(Q_Fit_) for June 1, 1957. (TIF)

S5 Fig Cluster map of p(Q_Fit_) for June 1, 1967. (TIF)

S6 Fig Cluster map of p(Q_Fit_) for June 1, 1974. Red squares indicate industrial sites that are the foci of significant case clustering at the 5% of smaller level. The group of statistically significant focused clusters in the southwest are in the city of Jackson, Michigan. At this time there was significant focused clustering when all of the industrial sites were considered simultaneously (significant global focused clustering).
(TIF)
