# Supplementary figures and images for: Local Bladder Cancer Clusters in Southeastern Michigan Accounting for Risk Factors, Covariates and Residential Mobility
Source: PLoS One. 2015 Apr 9;10(4):e0124516. doi: 10.1371/journal.pone.0124516 (PMC4391784; doi:10.1371/journal.pone.0124516)

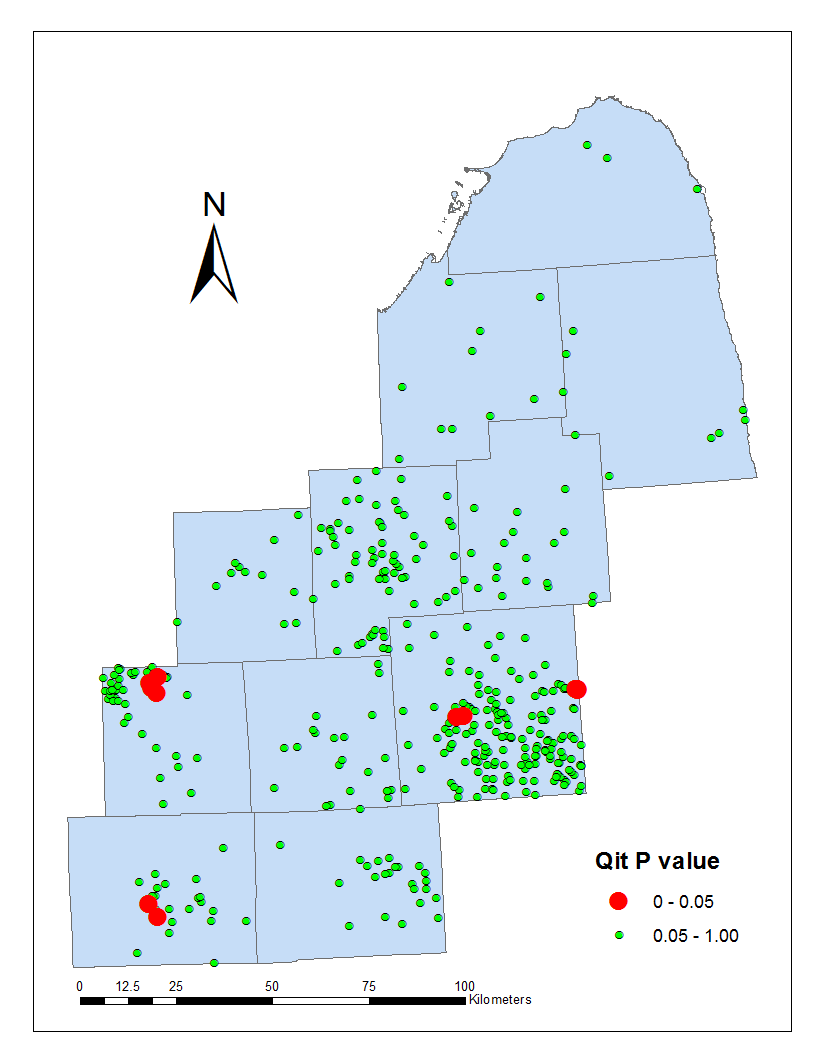

Supplement: S1 Fig — Maps of the significant local space-time statistic p(Qit) illustrate the persistent local bladder cancer clusters in Oakland county, Ingham county near Lansing, and in the city of Jackson. Visualized for October 1, 1998. (TIF) [file pone.0124516.s001.tif]

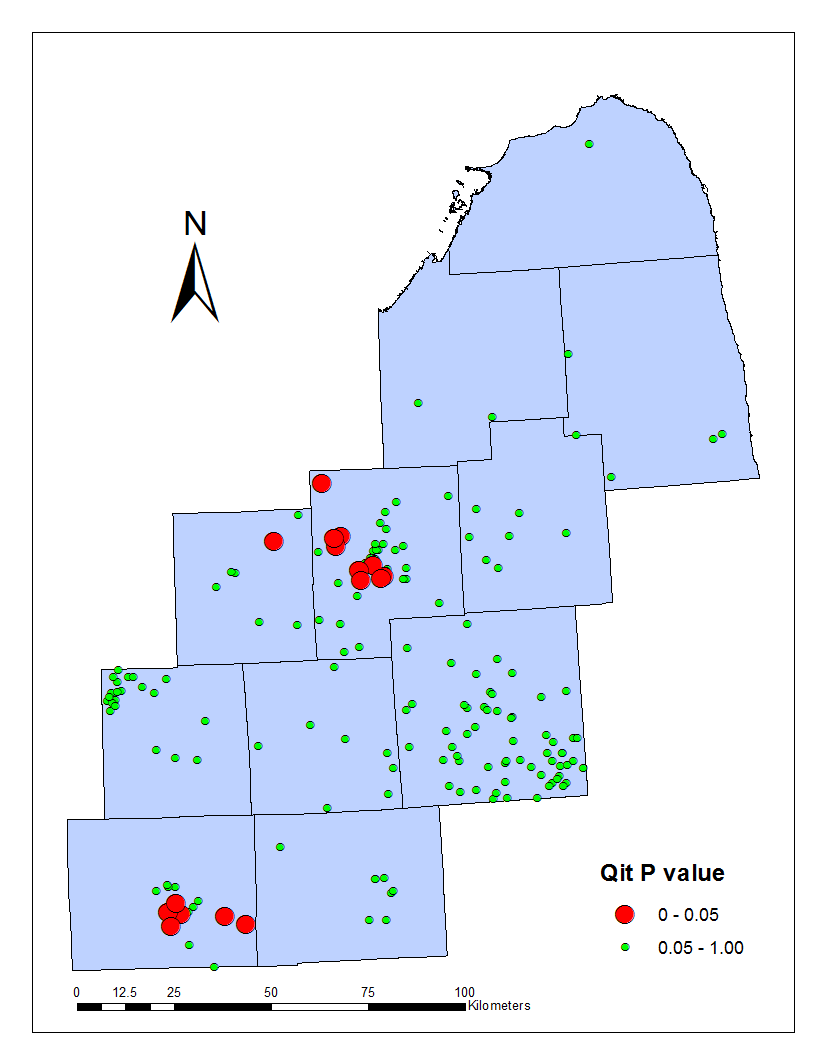

Supplement: S2 Fig — The Jackson cluster constructed using place of residence of cases and controls at age 33 years. (TIF) [file pone.0124516.s002.tif]

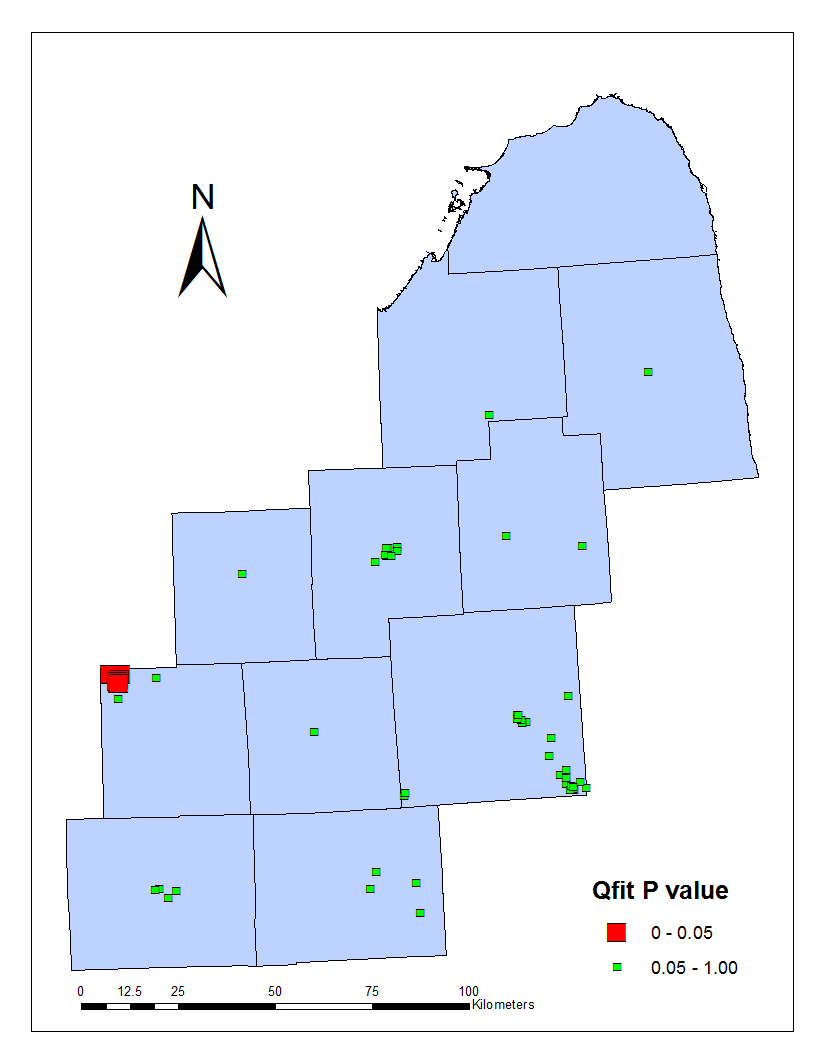

Supplement: S3 Fig — Red squares indicate industrial sites that are the foci of significant case clustering at the 5% of smaller level. The group of significant foci in the Lansing area are responsible for the large decrease in the global cluster statistic QF that began in 1956 and 1957, and persisted through 1964. By 1967 this cluster was no longer significant. (TIF) [file pone.0124516.s003.tif]

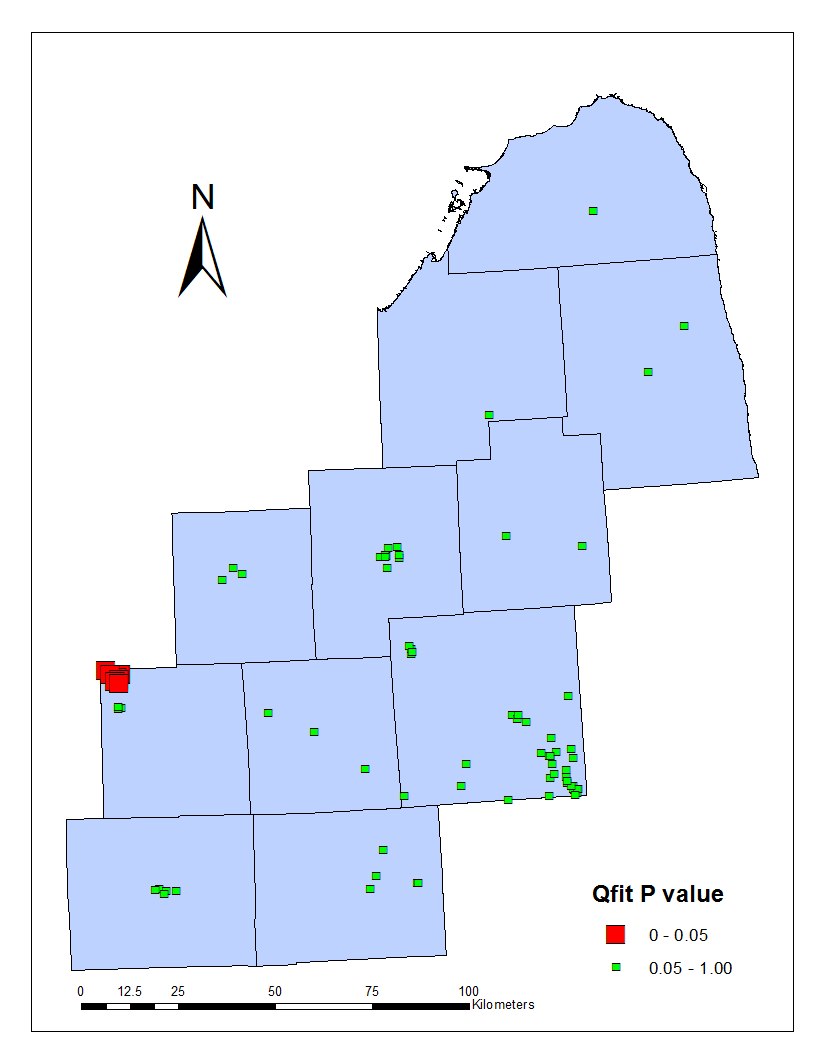

Supplement: S4 Fig — (TIF) [file pone.0124516.s004.tif]

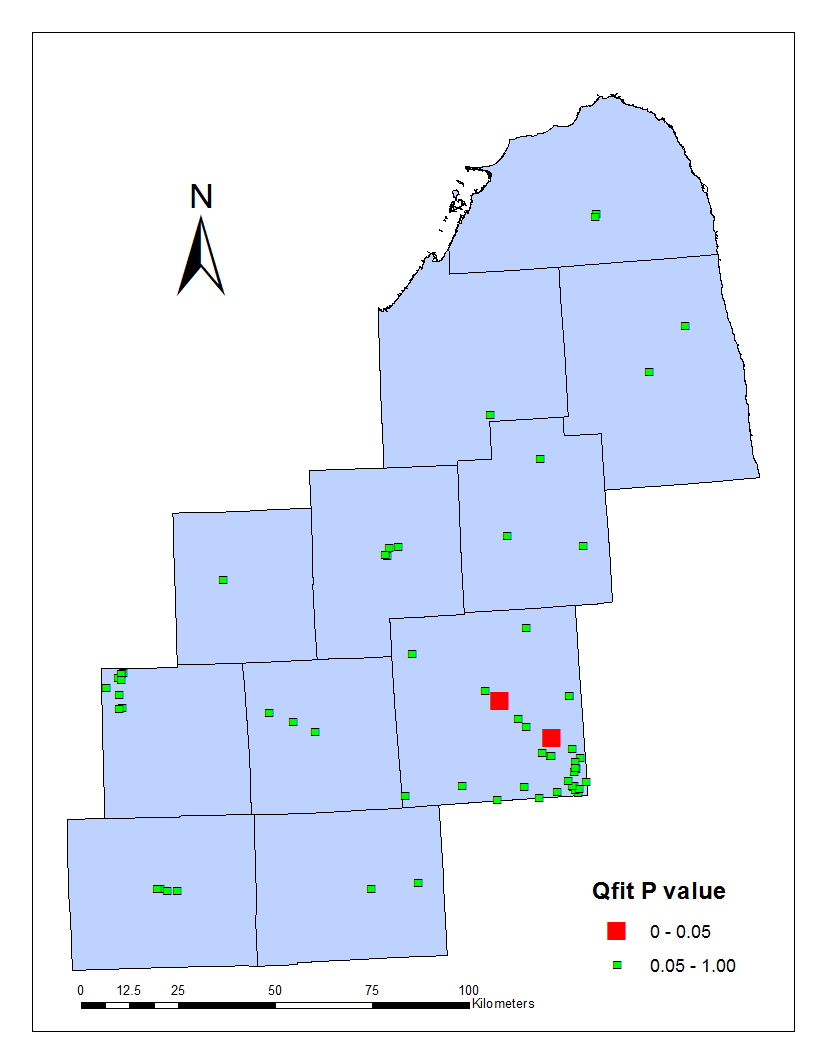

Supplement: S5 Fig — (TIF) [file pone.0124516.s005.tif]

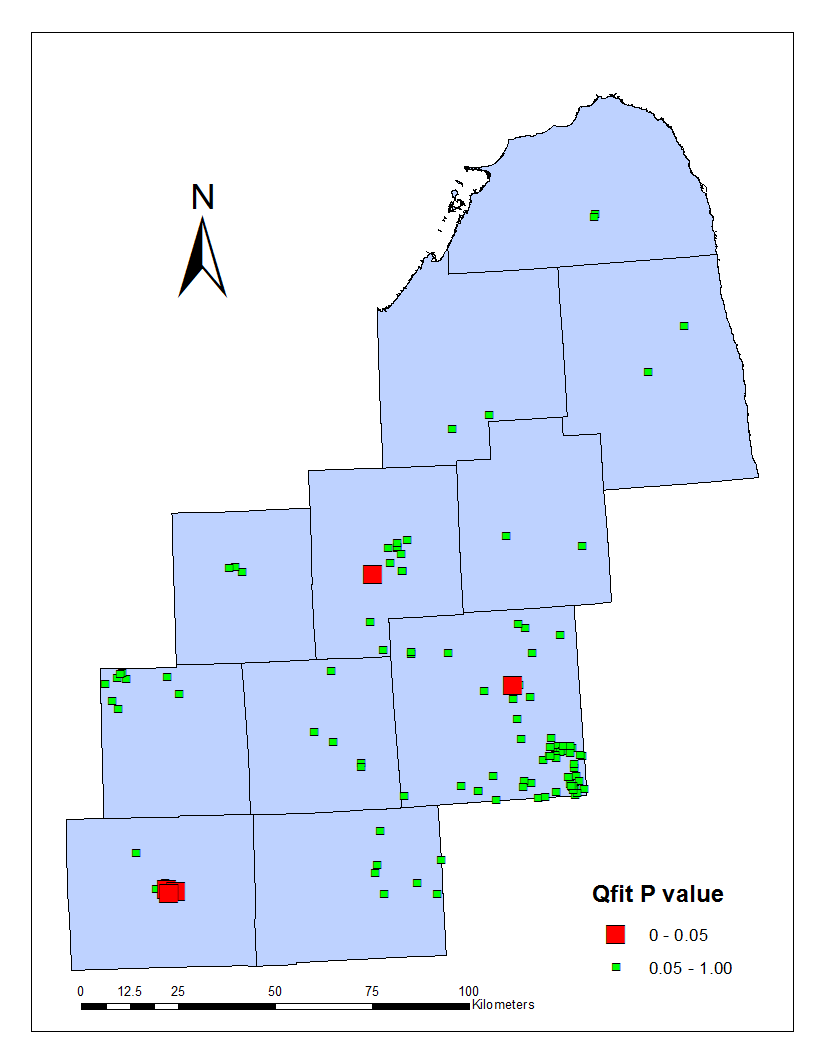

Supplement: S6 Fig — Red squares indicate industrial sites that are the foci of significant case clustering at the 5% of smaller level. The group of statistically significant focused clusters in the southwest are in the city of Jackson, Michigan. At this time there was significant focused clustering when all of the industrial sites were considered simultaneously (significant global focused clustering). (TIF) [file pone.0124516.s006.tif]
